# Single-Cell and Spatial Omics Technologies in Rice Abiotic Stress Biology: A Methodological Review

**DOI:** 10.3390/ijms27167114

**Published:** 2026-08-08

**Authors:** Junxiao Chen, Zheng Chen, Chun Yin, Lei Zhou, Da Zhao

**Affiliations:** 1 Institute of Food Crops Research, Hubei Academy of Agricultural Sciences, Wuhan 430064, China; cjx881112@163.com (J.C.); chenzheng7@126.com (Z.C.); jhdmym@163.com (C.Y.); 2 Fujian Key Lab of Agriculture IOT Application, School of Information Engineering, Sanming University, Sanming 365004, China

**Keywords:** *Oryza sativa*, single-cell RNA-seq, spatial transcriptomics, single-cell multi-omics, DNA methylome, abiotic stress, methodology, evidence grading, deep learning

## Abstract

Abiotic stresses—drought, salinity, extreme temperature, flooding, and heavy-metal toxicity—constrain rice (*Oryza sativa* L.) yield worldwide, and the cellular programmes underlying them are unevenly distributed across cell types that bulk-tissue assays average together. This review examines, from a methodological standpoint, what single-cell and spatial omics technologies can and cannot establish about rice abiotic stress biology. We first define the modality space: single-cell omics measures RNA, chromatin accessibility, DNA methylation, protein, or metabolite features at the resolution of individual cells or nuclei, whereas spatial omics measures such features while retaining tissue coordinates; the two are complementary rather than interchangeable. We then treat each platform class—droplet-based scRNA-seq, combinatorial-indexing approaches including SPLiT-seq, nuclei-based snRNA-seq and multiome, sequencing-based and imaging-based spatial transcriptomics—under a common template covering measurement principle, the questions each can answer, applicability to rice tissues, dominant biases, and the inferences each cannot support. To make evidence strength comparable across a heterogeneous literature, we apply a four-tier scheme throughout: Tier A, direct rice cell-resolved or spatial evidence with functional or field validation; Tier B, robust rice functional and localization evidence without single-cell data; Tier C, cell-resolved evidence without causal validation; and Tier D, cross-species analogy or reasoned proposal. Applying this scheme shows that the genes with genuine breeding traction in rice—*SUB1A*, *OsHKT1;5*, *OsHMA3*, *OsNRAMP5*, *DRO1*—rest on Tier B evidence from classical genetics and field testing, whereas the most cell-resolved rice evidence concentrates in root outer layers and barrier formation at Tier C, and heat and cold stress, despite dominating yield loss, lack rice cell-resolved data almost entirely. We extend the discussion beyond transcriptomics to single-cell DNA methylome profiling, spatial proteomics and metabolomics, and three-dimensional analysis of thick plant tissues, in each case distinguishing demonstrated plant capability from mammalian-only capability, and we assess the expanding role of artificial intelligence in annotation, segmentation, batch correction, integration, and perturbation prediction alongside its documented failure modes. Rice, maize, and wheat are compared to identify transferable methodology. Cell-resolved omics has to date improved biological interpretation and candidate prioritization; demonstrating an incremental breeding advantage from it remains an unmet requirement.

## 1. Introduction

Rice (*Oryza sativa* L.) is the staple food crop for more than half of the global population, contributing about 80% of dietary calories for roughly half of humanity, with more than 90% of production and consumption concentrated in developing countries [1]. This concentration makes rice yield disproportionately vulnerable to climate change, soil degradation, and extreme weather [2]. The magnitude is quantifiable: long-term IRRI records show grain yield declining by about 10% for each 1 °C rise in growing-season minimum temperature [3], and four independent modelling approaches converge on a mean 3.2% reduction in global rice yield per 1 °C of warming [4].

Plants do not respond to abiotic stress through uniform whole-plant programmes: root epidermis, exodermis, endodermis, vascular parenchyma, leaf mesophyll, guard cells, and anther tapetum differ profoundly in how they perceive, transduce, and execute stress responses. Bulk transcriptomics, proteomics, and metabolomics identified central resilience genes such as *OsHKT1;5*/*SKC1* [5] and *SUB1A* [6], but average across cell types, masking low-abundance signals and failing to resolve which cells act, where, and when.

Because terminology in this field is used inconsistently, we fix it at the outset. Single-cell omics denotes measurement of molecular features—RNA transcripts, chromatin accessibility, DNA methylation, proteins, or metabolites—at the resolution of individual cells or nuclei, with cell identity preserved through barcoding or physical isolation, whereas spatial omics denotes measurement of such features while retaining the coordinates of the tissue from which they derive. These are orthogonal properties rather than points on one axis: a method may resolve single cells without preserving position (scRNA-seq), preserve position without resolving single cells (spot-based spatial transcriptomics), or achieve both (imaging-based in situ methods). Single-cell transcriptomics and single-cell omics are therefore not synonyms, and we use the former where only RNA has been measured. The full modality space—scRNA-seq and snRNA-seq, single-cell chromatin accessibility (scATAC-seq), joint RNA-and-chromatin profiling (multiome), single-cell DNA methylome sequencing, single-cell proteomics, and the spatially resolved counterparts of several of these, together with spatial metabolomics by mass spectrometry imaging—is at radically different stages of maturity in plants, and one purpose of this review is to state those stages accurately rather than to present the list as though it were uniformly available. The review is accordingly centred on single-cell and spatial transcriptomics in rice, where the substantive literature exists; it extends to chromatin accessibility, now routinely co-profiled, to single-cell DNA methylation, established in mammals and absent in plants, and to proteomic and metabolomic layers treated as complementary evidence that can corroborate or contradict transcriptomic inference rather than as parallel fields to be surveyed. Throughout, we are concerned with what each method establishes and treat breeding applications as a downstream question of whether that evidence is strong enough to act on.

Comparing evidence across this literature is difficult because studies differ in species, platform, treatment, and validation depth, and narrative review text tends to flatten those differences. We therefore apply a consistent four-tier scheme in the tables and text below. Tier A denotes direct cell-resolved or spatial evidence obtained in rice and supported by functional or field validation of the same gene or process; Tier B, robust functional and localization evidence in rice (mutant phenotype, protein localization, cloned QTL, field trial) but without single-cell or spatial data underpinning the cell-type assignment; Tier C, cell-resolved or spatial evidence in rice without causal or functional validation; and Tier D, evidence from another species or platform, or a reasoned methodological proposal not yet tested in rice. The scheme is deliberately coarse, and its purpose is to prevent the common conflation of “most cell-resolved evidence available” with “closest to application,” which, as Section 4 shows, are close to inversely related in the current rice literature.

Over the past five years, scRNA-seq [7], snRNA-seq and multiome [8], scATAC-seq [9,10], combinatorial-indexing methods [11,12], and spatial transcriptomic platforms [13,14,15] have been deployed across Arabidopsis, rice, maize, and other plants [16,17,18,19]. In rice, foundational studies include the 2021 root tip atlas [20], the 2021 multi-tissue multi-stress atlas of 237,431 cells [21], cell-type-specific gene regulatory-network analysis under water extremes [22], and the 2025 multi-organ multi-omics atlas [23], the published rice single-cell and spatial studies and their study-design attributes are summarized in Table 1. Section 2 and Section 3 treat platforms and rice atlases as the methodological core, with a cross-crop comparison against maize and wheat; Section 4 presents each stress as a methodological case study; Section 5 covers spatial omics including three-dimensional approaches; Section 6 addresses multi-omics integration beyond transcriptomics; Section 7 treats translational implications; and and Section 8 addresses the field’s obstacles, such as data integration, standardization, and the emerging role of artificial intelligence.

## 2. Technology Platforms: Measurement Principles, Applicability, and Inferential Limits

**Figure 1:**
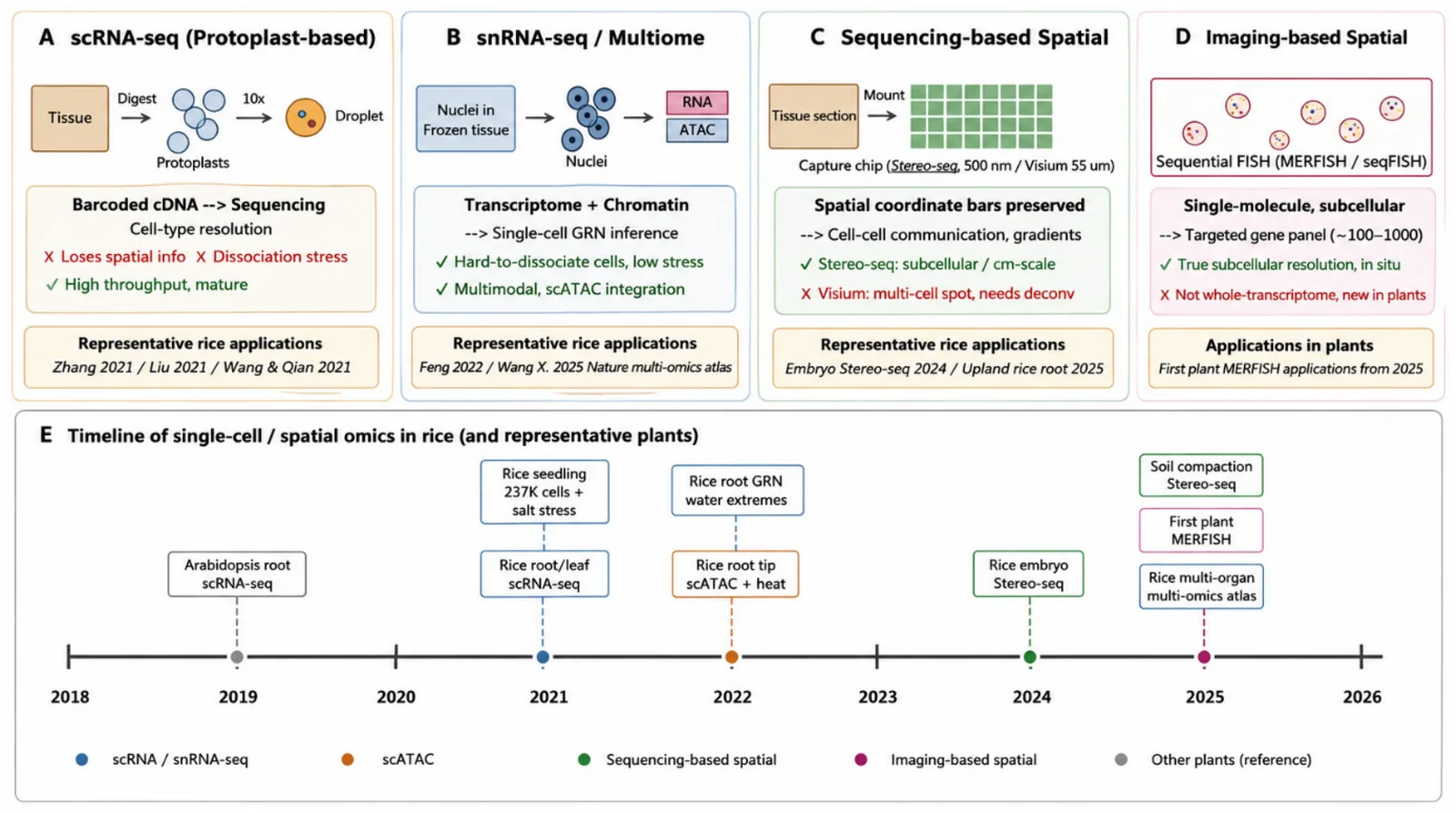
Single-cell and spatial omics technology principles and rice timeline. (**A**) scRNA-seq enzymatically digests tissue into protoplasts before loading into droplet microfluidics; strengths are high throughput and a mature workflow, weaknesses are loss of spatial information and dissociation-induced artifacts; representative rice applications are the root and seedling atlases of Zhang et al. 2021 [20], Liu et al. 2021 [24], and Wang and Qian 2021 [21]. (**B**) snRNA-seq and multiome use isolated nuclei and can co-profile transcriptome and chromatin accessibility, suiting hard-to-dissociate cells and stress-sensitive material, as in the rice root scATAC-seq study of Feng et al. 2022 [32] and the multi-organ multi-omics atlas of Wang et al. 2025 [23]. (**C**) Sequencing-based spatial omics (Stereo-seq, Visium) preserve spatial coordinates and suit studies of radial gradients and cell–cell communication, as in the rice embryo germination atlas of 2024 [33] and the upland-rice root study of 2025 [34]. (**D**) Imaging-based spatial omics (MERFISH, seqFISH+) achieve subcellular single-molecule resolution by sequential in situ hybridization [35,36]; application to plants remains at an early stage, and no established rice application yet exists. (**E**) Key milestones for rice and reference plants, comprising the Arabidopsis root scRNA-seq benchmark [16], the rice root and seedling atlases [20,21,24], the rice root GRN analysis under water extremes [22], the rice root-tip scATAC-seq heat study [32], the rice embryo Stereo-seq atlas [33], the soil-compaction spatial study [28], and the rice multi-organ multi-omics atlas [23]. Combinatorial-indexing methods share the library-chemistry principle of (A) while replacing droplet compartmentalization with iterative split-pool barcoding [11].

The platforms considered in this review differ along four axes that jointly determine what a given experiment can establish: whether cell identity is resolved, whether tissue position is retained, which molecular layer is measured, and how many cells, conditions, and replicates are affordable. Confusion in the literature usually arises when a conclusion licensed by one axis is drawn from a platform that is strong on a different one. A dissociation-based transcriptomic experiment cannot establish where in a tissue a response occurs, however finely it resolves cell identity; a spot-based spatial experiment cannot establish which cell type responded, however precisely it localizes signal. In describing each platform below, we therefore give equal weight to what it measures and to the inferences it cannot support, and Table 2 condenses these limits into explicit “conclusions supported” and “conclusions not supported” columns, since these are what determine whether a published claim is adequately underpinned. Figure 1 places the platform families and their principal rice milestones on a common timeline.

### 2.1. Dissociation-Based Transcriptomics: Droplet, Combinatorial-Indexing, and Plate-Based Methods

Droplet-based scRNA-seq is the most mature single-cell platform and the one on which the first wave of rice studies was built. Tissue is enzymatically digested to release protoplasts, which are co-encapsulated with barcoded beads in nanolitre droplets for reverse transcription and library construction, with cell identity established by droplet compartmentalization and throughput reaching 10^4^–10^5^ cells per run [7]. On this basis, the early rice atlases established root and seedling cell-type inventories, defining which transcriptionally distinct populations exist in a tissue, how their abundance and expression state differ between conditions, and, through trajectory inference, how populations relate along developmental continua [20,21,24]. The approach is best suited to root tips, young leaves, and whole seedlings, where dissociation is tractable, and poorly suited to mature stems, lignified tissue, and field-collected material. Three biases interact in ways that are especially consequential for stress work. The one-to-two-hour enzymatic digestion is itself a stress treatment, inducing heat-shock proteins, wounding responses, and immune-related genes whose signature overlaps substantially with genuine abiotic stress responses—a more serious confounder here than in developmental studies, and one that makes a protocol-matched unstressed control indispensable [16,17]. Cell-type recovery is non-uniform, because rice exodermis, cortex, and vascular parenchyma differ markedly in digestion susceptibility, so the recovered composition is not tissue composition. And protoplasting destroys position entirely. These biases are not independent: the cell types most resistant to dissociation, notably mature vascular and lignified cells, are also those most involved in long-distance ion transport, so the recovery bias systematically depletes the cells of greatest interest for salinity and heavy-metal work. Droplet scRNA-seq consequently cannot, on its own, establish that a response occurs at a specified tissue position, that observed cell-type proportions reflect tissue composition, or—without a matched control—that a stress-associated signature is not a dissociation artefact.

Combinatorial-indexing methods dispense with physical compartmentalization altogether, and their relevance to rice stress biology is a matter of experimental design rather than of chemistry alone. In SPLiT-seq, fixed cells or nuclei are distributed across a multiwell plate, barcoded in situ by ligation, pooled, redistributed, and barcoded again; after several rounds the probability that two cells share the full barcode combination becomes negligible, so cell identity emerges combinatorially rather than from a droplet [11], and the related sci-RNA-seq family applies the same logic with different barcoding chemistry [12]. Because barcoding requires only plates and standard liquid handling rather than proprietary microfluidics, cost per cell falls and, more importantly, many samples can be processed together within one experiment. This directly addresses the central methodological weakness of the rice stress single-cell literature documented in Section 8—that studies use one or two genotypes, one stress intensity, and minimal replication because per-run costs are high—since fixed material from many genotypes, stress gradients, timepoints, and replicates can be barcoded together with sample identity encoded in the first barcoding round. For a question such as whether tolerant and susceptible cultivars differ in the cell-type distribution of a salt response, this breadth matters more than per-cell sensitivity. The trade-off is real and quantified: in the one plant demonstration, applying combinatorial-indexing snRNA-seq and snATAC-seq across rice and sorghum to compare C3 and C4 cell-identity networks, a droplet platform recovered approximately 50% more unique molecular identifiers per nucleus than combinatorial indexing [32]. Combinatorial indexing thus buys experimental breadth at a cost in per-cell depth, producing sparser data that weakens annotation of rare and transitional populations and reduces power to detect low-abundance transcription factors—precisely the genes regulatory analyses target—while requiring a fixation step not yet well optimized for plant tissue, and it neither confers spatial information nor corrects cell-recovery bias, instead reproducing that bias consistently across many samples. A third family, the plate-based full-length methods, occupies a distinct niche: Smart-seq2, Smart-seq3, and CEL-Seq2 sequence far fewer cells but capture full-length transcripts, enabling the isoform- and allele-resolved analysis that no barcoded 3′-end method provides [33,34,35]. Because rice stress responses involve alternative splicing and, in hybrids and heterozygotes, allele-specific expression, this niche is not displaced by higher-throughput platforms. The three families are therefore complementary rather than competing—droplet methods maximizing per-cell depth at moderate throughput, combinatorial indexing maximizing sample multiplexing, and plate-based methods maximizing transcript information per cell.

### 2.2. Nuclei-Based Methods and Multiome

Single-nucleus methods release nuclei by mechanical homogenization rather than enzymatic digestion, then process them through droplet or combinatorial-indexing chemistry, and the multiome configuration extends this by co-assaying transposase-accessible chromatin and RNA within the same nucleus, as applied across eight rice organs and 116,564 cells [8,23]. Beyond the population-level questions that scRNA-seq answers, multiome additionally reports which regulatory regions are accessible in which cell type, allowing inference of cell-type-specific regulatory networks and nomination of candidate promoters and cis-regulatory elements. The choice between whole-cell and nuclear input is not a matter of preference but changes which cells are observed and, potentially, which conclusion is reached. Nuclei extraction improves recovery of mature, lignified, and hard-to-dissociate cells and tolerates flash-frozen material, making it the only practical route for field-collected samples and for cold-stress work, where warm enzymatic digestion of cold-treated tissue would confound the treatment. Against this advantage, nuclear transcriptomes capture fewer transcripts per cell and are enriched for intron-containing nascent RNA. The consequence is more than quantitative: because detection power for lowly expressed regulators falls, a transcription factor identified as cell-type-specific in whole-cell data may be undetectable in nuclear data from the same tissue, and cytoplasmically stable transcripts are systematically under-represented relative to nascent ones. Divergence between scRNA-seq and snRNA-seq results in rice therefore reflects both technical bias and a genuine difference in the molecular pool sampled, and cross-study comparisons that ignore the distinction can misattribute a methodological difference to biology. Multiome strengthens candidate nomination but cannot establish that an accessible chromatin region is transcriptionally active, or that accessibility differences are causal for a phenotype; accessibility indicates regulatory potential only.

### 2.3. Spatial Transcriptomics: Sequencing-Based and Imaging-Based Platforms

Sequencing-based spatial transcriptomics recovers the position that dissociation discards, by mounting a tissue section on a substrate bearing spatially barcoded capture probes so that transcript coordinates are recorded at capture [13]. Platforms differ chiefly in feature size—Stereo-seq achieves sub-micron resolution across centimetre-scale fields using DNA nanoball arrays [15], Slide-seq reaches roughly 10 µm [14], and Visium uses 55 µm spots—and this resolution, relative to the anatomy in question, is the variable that determines what can be concluded. Rice roots are an unusually demanding case: a 55 µm Visium spot spans epidermis, exodermis, and outer cortex, the very layers a salinity or barrier study seeks to distinguish, so Visium data in rice roots support tissue-level but not cell-layer-level claims unless deconvolution against a matched single-cell reference is performed, in which case the claim inherits that reference’s recovery biases. Stereo-seq resolves these layers directly, which is why the informative rice spatial studies to date have used it, in *Arabidopsis* leaf and in rice embryo germination and upland-root adaptation [28,36,37]. Section orientation determines which anatomical relationships are visible at all, capture efficiency varies across a section, and per-bin transcript counts at sub-micron resolution are low enough that binning is required—reintroducing a resolution choice made analytically rather than experimentally. These methods cannot demonstrate that co-localization of a signalling gene and a response gene reflects signal transfer between them, cannot attribute a spot-level signal to a particular cell type without independent deconvolution, and cannot warrant that a gradient seen in one section generalizes across the organ.

Imaging-based spatial transcriptomics reaches the highest spatial precision by performing sequential rounds of in situ hybridization with imaging readout, assigning transcripts to subcellular positions within intact tissue and using error-robust barcoding to multiplex on the order of 10^3^ genes [38,39], with in situ sequencing variants extending the approach into intact three-dimensional volumes [40]. Where a small, pre-selected panel of transcripts must be localized to a specific cell type or subcellular compartment, these methods reach a certainty that dissociation- and capture-based approaches cannot. Their adaptation to plants, however, lags well behind animal applications: cell walls impede probe penetration, autofluorescence from lignin and phenolics degrades signal-to-noise, and large vacuoles displace cytoplasm into thin peripheral layers that complicate segmentation; while clearing chemistry developed for plants mitigates these problems, it does not eliminate them [41]. In rice, the practical consequence is that imaging-based spatial transcriptomics is at present a confirmatory tool, appropriate for verifying the localization of a handful of nominated candidates rather than for discovery, and because the panel must be specified in advance it encodes prior hypotheses and cannot generate unbiased discovery; any claim that it is routinely available for plant tissue would be premature.

### 2.4. The Analytical Pipeline and Its Inferential Pitfalls

The analytical pipeline shared across these platforms—quality control, dimensionality reduction, clustering, batch correction, annotation, differential expression, and trajectory inference [42,43,44,45,46], supported for plants by marker resources such as scPlantDB, PsctH, and PlantscRNAdb [47,48,49], with deconvolution and reference mapping added for spatial data [50,51,52]—contains two choices whose consequences are large enough to change conclusions. The first is the unit of statistical replication. Testing differential expression across individual cells treats cells as independent samples, which they are not, since cells from one plant share its genotype, treatment history, and handling batch; this inflates significance dramatically, and benchmarking in other systems shows that pseudo-bulk aggregation per cell type per sample controls false discovery far better [53]. Because most rice stress studies have only one or two biological replicates, the choice is often forced rather than free, and reported differential-gene counts across studies are consequently not comparable. The second is annotation: cell-type labels in rice are assigned largely from marker genes derived from a small number of reference studies, so annotation errors propagate silently across the literature, and the cell types lacking canonical rice markers—the multilayered cortex, lysigenous aerenchyma, and transitional states—are the ones most likely to be mislabelled.

## 3. Rice Cell-Type Atlases as a Reference Coordinate System

**Figure 2:**
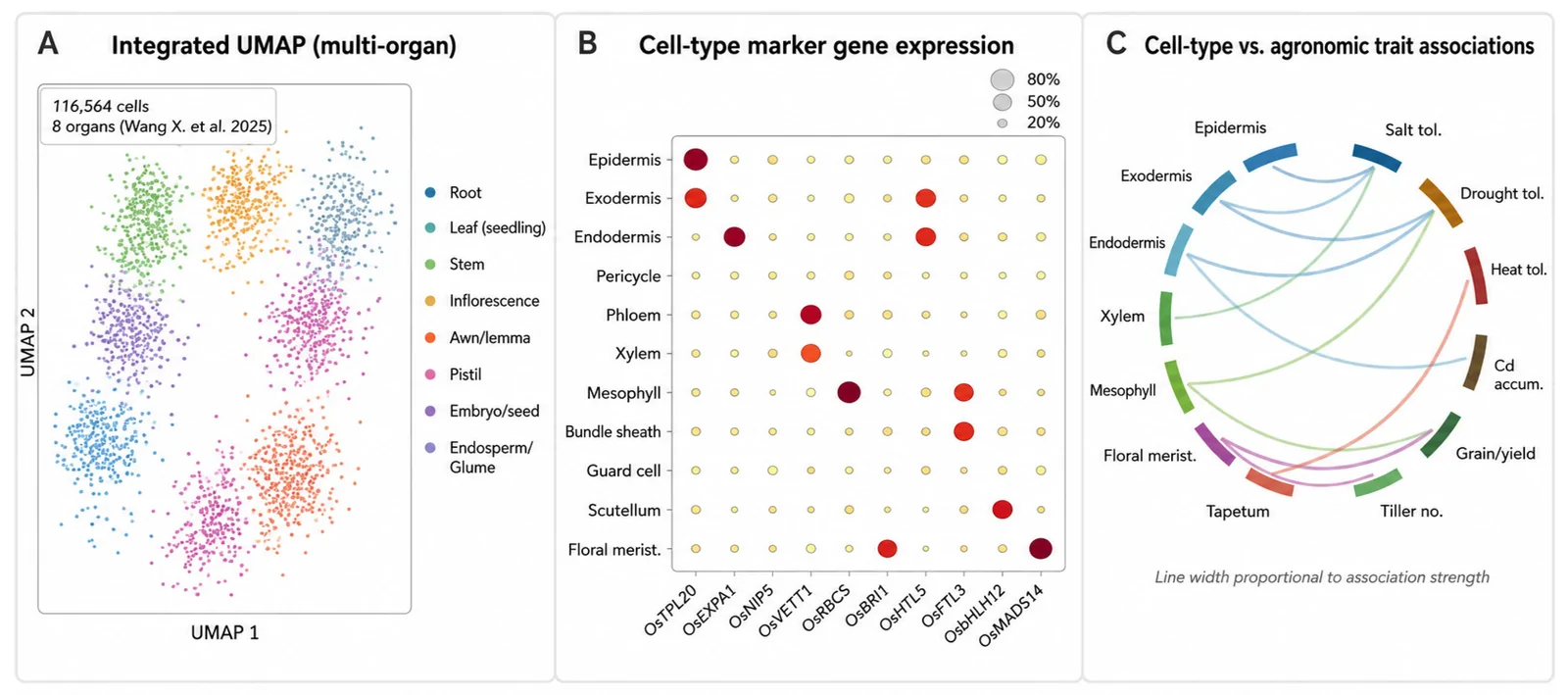
Integrated overview of the rice multi-organ single-cell atlas. (**A**) Integrated UMAP of cells from eight organs, with cells coloured by organ of origin (see in-panel legend). (**B**) Dot plot of cell-type-specific marker gene expression, in which dot colour encodes the mean scaled expression level of each gene (from yellow, low, to dark red, high) and dot size encodes the percentage of cells within the cell type expressing the gene (see size legend). (**C**) Chord diagram of cell-type-by-agronomic-trait associations, line width indicating association strength. Schematic re-drawn from Wang X. et al. 2025 [23].

A cell-type atlas is the reference frame against which every subsequent stress observation is interpreted, converting an expression change from an organ-level statement into a statement about a specific cell population, its lineage, and its regulatory context. For methodological purposes, the atlas functions as a lookup table: given a gene nominated by mapping or bulk analysis, it returns the cell types expressing that gene and the developmental stage at which they do so, which in turn constrains whether the gene is a plausible mediator of a trait and where an intervention would have to act. The paragraphs below survey the rice atlases as such a reference, organ by organ, and then compare their coverage against maize and wheat to identify what is transferable and what is rice-specific (Figure 2).

The rice root is the most thoroughly resolved organ. The root tip atlas of Zhang et al. profiled roughly 27,000 cells across nine major cell types with paired chromatin accessibility [20], and Liu et al. independently resolved the meristem-to-maturation trajectory [24]. Two rice-specific anatomical features that distinguish the rice root from the *Arabidopsis* reference—a multilayered cortex and lysigenous aerenchyma [18]—are captured in current data, which is what makes this atlas usable as the coordinate frame for the major root-borne stresses: it identifies the layer that excludes, sequesters, or unloads Na^+^ under salinity, the layer in which apoplastic barriers form under water deficit, the sites at which Cd, As, and Mn enter and are retained, and the cortical cells in which aerenchyma forms under waterlogging. Its principal limitation is developmental rather than technical, since nearly all of these data derive from seedling root tips grown hydroponically, which leaves mature zones, crown and lateral roots, and soil-grown roots—the tissues that ultimately determine field performance—essentially unrepresented.

The leaf and seedling are anchored by the Wang and Qian atlas of 237,431 cells, which resolved 15 leaf and nine root cell types and, uniquely among the large rice datasets, included NaCl and low-nitrogen treatments, making it the primary large-scale rice stress single-cell resource [21]. Bundle-sheath-specific transcription factors identified subsequently add regulatory references relevant to engineering C4-like traits [25]. This atlas serves as the reference frame for photosynthetic thermotolerance, stomatal regulation and water-use efficiency, source–sink loading, and senescence timing, but its usefulness for those questions is qualified by two gaps: guard cells and other low-abundance epidermal types are under-recovered in protoplast data, and no rice leaf dataset yet covers heat or drought at the reproductive stage, when yield is most sensitive.

The inflorescence, anther, and pistil are covered by the young-panicle atlas, which resolved floral and spikelet meristems and stamen primordia [26], by snRNA-seq resolution of pistil trajectories [27], and by the addition of anther and pistil to the common multi-organ reference [23]. Together, these provide the cell-type context for high-temperature spikelet sterility—specifically the tapetal programmed cell death underlying pollen abortion—and for seed set and grain number, and they are what would be needed to interpret cloned heat-tolerance loci such as *qHTSF4.1* [29] at cell resolution. That interpretation cannot yet be made, however, because no published rice anther or pistil dataset includes any stress treatment, which is precisely why reproductive-stage heat, the dominant cause of heat-related yield loss, remains the least cell-resolved of the major stresses.

The embryo and seed are represented by a Stereo-seq study that, combined with single-cell profiling, produced a spatiotemporal atlas of germination and identified two previously unrecognized scutellum cell types [28]; this bears on seed vigour and establishment under cold or saline direct seeding, where anaerobic germination is governed by *OsTPP7* [30]. The most systematic resource across organs is the multi-organ multiome atlas of 116,564 cells, which built cell-type-specific regulatory networks and, in the step most consequential for applied work, intersected cell-type accessibility with rice GWAS signals to produce cell-type-by-trait associations [23]. Its companion *Poaceae* atlas of 103,911 nuclei across five species and 126 cell states [31] extends the comparative regulatory frame established in maize [10] and the pan-grass transcriptome [19]. All of these are developmental references from single control-condition genotypes, so the cell-type-by-trait associations they yield are associations with chromatin accessibility rather than demonstrations of causality, and they inherit the population and environment limitations of the underlying GWAS. For rapid interrogation of these resources, curated databases—scPlantDB, integrating roughly 2.5 million cells from 67 datasets across 17 species [47]; PsctH, curating protoplasting protocols and markers [48]; and PlantscRNAdb, covering markers across 33 species [49]—provide the fastest route to check the cell-type expression profile of a gene without reanalyzing raw data, subject to the caveat that their marker sets inherit whatever annotation errors the source studies contained.

Read across organs, atlas coverage is markedly uneven, and the unevenness maps inversely onto breeding priority: root coverage is richest but confined to seedling tips, leaf coverage uniquely includes stress treatments but under-samples the guard cells central to water-use efficiency, and reproductive and embryo atlases exist but contain no stress treatment at all. The organ with the most cell-resolved data, the seedling root, is therefore not the organ where the largest yield-limiting stresses act, which are reproductive-stage heat and drought, so matching atlas coverage to breeding need would prioritize reproductive-organ stress atlases and mature, field-grown root tissue, both currently near-absent.

Placing rice alongside the other two major cereals clarifies which of these obstacles are intrinsic to rice and which methodological solutions can be borrowed. Maize single-cell work is comparably mature—a multi-organ single-cell regulatory atlas spans root, seedling, and reproductive tissues [10], leaf studies have resolved bundle-sheath phloem loading [54], and, importantly for stress biology, a maize root heat-stress study using single-cell transcriptomics identified the cortex as the principal heat-responsive cell type [55]. That result is one of the few cereal abiotic stress single-cell studies with a clear cell-type conclusion, and it converges with the rice root scATAC-seq heat result [56]; the convergence is consistent with a conserved cortical heat response across *Poaceae*, though, as discussed below in the treatment of heat stress, the differing species, platforms, and readouts mean it does not amount to proof of conservation. Wheat provides the instructive contrast: its roughly 17 Gb allohexaploid (AABBDD) genome makes protoplast-based work and homoeolog-resolved annotation substantially harder, and the field has consequently leaned on single-nucleus strategies, with a single-nucleus multiome study of the bread wheat root resolving cell types and revealing asymmetric expression among homoeologous copies of the same gene [57], followed by a wheat root scRNA-seq developmental atlas [58]. The methodological lessons transfer in specific directions. From maize, rice work can borrow cross-species regulatory maps that indicate which cell-type programmes are conserved enough to inform candidate nomination; from wheat, it can borrow the single-nucleus and homoeolog-aware analytical strategies developed for a complex genome, which are directly relevant not to *japonica* references themselves but to applying atlases across the *indica*–*japonica* divide and to the polyploid wild relatives used as stress-tolerance donors. The rice-specific constraints—the exodermis and mature-root recovery problem, and the near-total absence of anther stress data—are solved by neither neighbour and require dedicated rice experiments (Table 3).

## 4. Stress Biology as Methodological Case Studies

**Figure 3:**
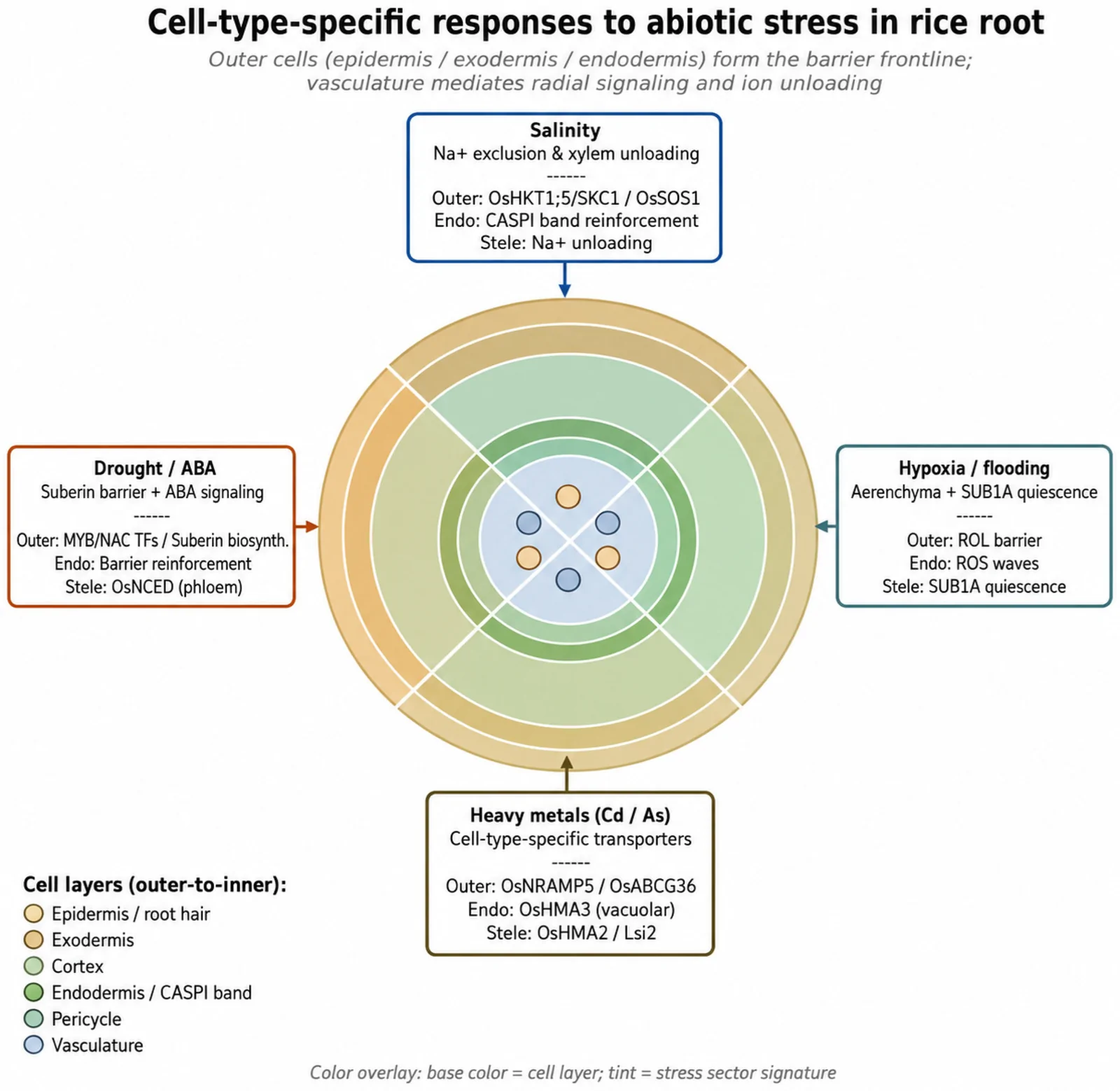
Cell-type-specific responses to abiotic stress in the rice root: a summary of published observations. The cross-section is divided into four stress sectors, each annotated with response genes reported for outer cells, endodermis, and stele. The figure collates the studies cited in this section and does not propose a new mechanistic model; genes differ substantially in the evidence tier supporting their cell-type assignment (Table 4).

Cell-resolved stress analysis separates three processes that are mixed in bulk tissue: stress perception, intercellular signalling, and execution of physiological responses. The following sections consider salt, drought, heat, cold, flooding, and heavy metals. For stresses entering through soil or water, the epidermis, exodermis, and endodermis often form the initial transport and barrier interface, while vascular tissues mediate radial and long-distance movement [3,4,5,6,7,8]. Figure 3 summarizes this root-specific interpretation.

### 4.1. Salt Stress: Na^+^ Exclusion and Unloading by Distinct Cell Types

The mechanistically central cell types are the stelar parenchyma, which retrieves xylem Na^+^ through *OsHKT1;5*/*SKC1* [5], the root epidermis, where *OsSOS1*-family antiporters efflux Na^+^ [59] and *OsHKT2;1* participates in K^+^/Na^+^ transport [60], and the endodermis and exodermis, whose suberin and lignin restrict apoplastic flow [61,62]. The strongest evidence here is nonetheless not cell-resolved: *OsHKT1;5* is a Tier B locus—a cloned QTL with transgenic and near-isogenic-line validation whose cell-type assignment comes from promoter-reporter work rather than any single-cell study [5]—while the clearest demonstration that cell-type-targeted expression can work, cortex-specific *AtHKT1;1* reducing shoot Na^+^ without a growth penalty, is Tier D evidence obtained in *Arabidopsis* [63] and not reproduced in rice. These two lines answer different questions and are not additive: the rice genetics establishes that the transporter matters, the *Arabidopsis* work that where it is expressed matters, and neither substitutes for a rice cell-resolved salt study. Such a study is conspicuously absent, since the one large rice salt dataset emphasized shared cross-stress patterns rather than salt-specific cell-type resolution [21] and the available root-hair-specific salt work exists only in *Arabidopsis* and *Brassica* [64]. A tolerant-versus-sensitive rice cultivar comparison by snRNA-seq under a controlled salt gradient—a design that combinatorial indexing now makes affordable—remains one of the field’s highest-value unperformed experiments.

### 4.2. Drought and ABA Signalling: Suberin Barrier and Radial Signalling

ABA is synthesized in the phloem-associated vasculature through *OsNCED* and *OsAAO1*, the exodermis and endodermis build suberin and lignin barriers under MYB and NAC control [65,66], root architecture is governed by *DRO1* [67], and *HMGB1* is enriched in the outer root cells of upland rice [37]. The strongest cell-resolved evidence in this case spans Tiers A to C in rice: Reynoso and Bailey-Serres resolved endodermal and exodermal suberin biosynthesis with regulatory-network support under water extremes [22], and Zhu, Hsu et al. showed spatially that ABA-biosynthesis genes are induced in phloem-associated vasculature under soil compaction, with the exodermis and endodermis showing the highest differential expression of any cell type [68], while *DRO1* contributes Tier B evidence with field-validated yield benefit [67]. Two boundaries must be kept explicit. The compaction result concerns soil compaction, a related but distinct stress from water deficit, so extrapolating its radial ABA pattern directly to drought is an inference rather than a finding; and, more strongly, the spatial data establish co-localization—ABA-biosynthesis transcripts in the vasculature and barrier-gene induction in the outer layers—so describing this as ABA “acting radially outward” would import a causal, directional claim that transcriptomic co-localization alone cannot support in the absence of hormone tracing, sensors, or perturbation. Stated precisely, the pattern is consistent with ABA moving from vascular-associated cells toward the outer layers. The *Arabidopsis* mesophyll dual-response pattern, in which one subpopulation prioritizes photoprotection and another osmotic adjustment [69], remains Tier D by species.

### 4.3. Heat Stress: Root Cortex and Anther Tapetum as Stress-Sensitive Tissues

The relevant cell types are the root cortex during vegetative growth and, at reproduction, the anther tapetum, microspore mother cells, and anther wall, with leaf mesophyll implicated in photosynthetic thermotolerance. The cloned loci—*TT1*, encoding a proteasome subunit that clears heat-damaged proteins [70]; *TT2*, acting through cuticular wax [71]; the *TT3.1*–*TT3.2* module protecting chloroplasts [72]; and the anthesis spikelet-fertility locus *qHTSF4.1* [29]—are all Tier B, functionally validated but none cell-type-resolved. The single-cell-resolved rice heat result is Tier C: root-tip scATAC-seq found cell-type-specific accessibility changes most pronounced in the cortex, unpaired with transcriptome or phenotype [56]. A maize root heat study independently found the cortex most differentially expressed [55], and it is tempting to read the two together as demonstrating a conserved cortical heat response; but they differ in species, in platform (chromatin accessibility versus transcriptome), in treatment, and in readout, so their convergence is consistent with cross-*Poaceae* conservation without being sufficient to prove it. The dominant methodological gap of the entire field sits here, because no rice anther heat-stress single-cell study exists, leaving the cell types responsible for the single most economically consequential heat trait—reproductive-stage spikelet sterility, which proceeds through premature or delayed tapetal programmed cell death and consequent pollen abortion—anatomically known but transcriptionally unprofiled under stress. snRNA-seq combined with spatial transcriptomics would resolve tapetum, microspore mother cells, and anther wall concurrently and is technically feasible now.

### 4.4. Cold Stress: From Known Genes to Cell-Resolved Insight

No rice cell types have been resolved at single-cell level, and the cloned genes of the ICE–CBF/DREB–COR pathway—*COLD1* [73], *HAN1* [74], *bZIP73* [75], and *CTB4a* [76]—are uniformly Tier B, well validated by classical genetics and with natural variation linked to chilling tolerance and temperate adaptation [74,75], but with cell-type distributions inferred from bulk data and subcellular localization rather than measured directly. No rice cold-stress single-cell study has been published. This is a methodologically avoidable gap rather than a technical impasse, because cold work is unusually well suited to snRNA-seq: flash-frozen sampling is compatible with the nuclei workflow and sidesteps the otherwise severe confound of applying warm enzymatic digestion to cold-treated tissue, which would itself trigger a temperature-shift response indistinguishable from the treatment of interest.

### 4.5. Flooding and Hypoxia: Aerenchyma Formation and SUB1A

The cortex forms lysigenous aerenchyma through programmed cell death, the outer layers form the radial oxygen loss barrier, and the nodes and internodes control elongation, with *SUB1A* mediating the quiescence strategy [6,77], *SNORKEL1/2* the deepwater escape [78], and *OsTPP7* anaerobic germination [30]. *SUB1A* is the review’s strongest case of deployable evidence—Tier B extended by large-scale field validation, having been cloned, introgressed into mega-varieties, and validated at scale [6,79]—while Reynoso et al. resolved the aerenchyma-associated depletion of cell-cycle transcripts and activation of cell-death executors at cell-type resolution, contributing Tier C evidence [22]. The instructive point for a methodological review is that *SUB1A*’s success predates single-cell technology entirely and was achieved without it, which bounds the claim that cell resolution is a prerequisite for breeding progress: it is an accelerant for difficult, poorly localized targets rather than a universal requirement.

### 4.6. Heavy-Metal Stress: Cell-Type-Specific Transporter Expression

Uptake occurs at the epidermis and exodermis, sequestration in the cortex and vacuoles, long-distance loading in the stele, and grain loading at the node, with *OsNRAMP5* mediating Cd and Mn uptake [80], *OsHMA3* sequestering Cd in vacuoles [81,82], *OsHMA2* loading Zn and Cd [83], *OsLsi1* and *OsLsi2* transporting Si and As with demonstrated polar localization [84,85], and *OsABCG36* effluxing Cd [86]. This case is distinctive not for the quantity of its single-cell data, which is small, but for the completeness of its evidence chain assembled from complementary modalities—mutant phenotype, protein immunolocalization, genetic effect, and released breeding material: *OsNRAMP5* editing produced low-Cd cultivars without yield penalty [87], *OsHMA3* is a cloned QTL with natural allelic variation [81], and *OsLsi1* and *OsLsi2* have polar localization shown directly by immunolocalization [84,85]. This Tier B evidence rests its cell-type assignments on protein localization rather than transcriptomics, which is a strength rather than a weakness and precisely the kind of orthogonal validation that single-cell transcriptomic candidates in other stresses still lack. The methodological lesson is that single-cell data are most powerful as one modality within such a chain, refining resolution, rather than as a standalone basis for causal claims—and indeed single-cell work here has so far refined the maps of transporters already known rather than nominating new causal genes, while the node grain-loading tissue that determines final grain Cd remains unprofiled at cell resolution.

Read across these cases, three patterns recur often enough to state as observations rather than as a model. The root outer layers—epidermis, exodermis, endodermis—are repeatedly among the most transcriptionally responsive cell types across salt, drought, compaction, and heavy-metal treatments [21,22,68], consistent with their position at the plant–soil interface; a recurring set of response modules appears in these layers, comprising cell-wall remodelling, transporter regulation, osmotic adjustment, and reactive oxygen species scavenging; and the WRKY, NAC, bZIP, and MYB transcription-factor families activate in the outer layers under multiple stresses. Two comparability cautions temper these observations. Their apparent generality partly reflects sampling, since the root tip is by far the most profiled tissue and its outer layers are anatomically over-represented; and the observation that one cell type activates similar gene sets across different stresses [21] could reflect either a genuinely shared programme or a common dissociation-plus-general-stress signature [16], a distinction that could only be settled by applying multiple stresses to one genotype under a common protocol—once more a design that combinatorial indexing newly enables. Above all, evidence tier and breeding proximity are close to inversely related: the genes with genuine traction (*SUB1A*, *OsHKT1;5*, *OsHMA3*, *OsNRAMP5*, *DRO1*) rest on Tier B classical evidence, whereas the richest cell-resolved rice evidence, in the outer-layer barrier programmes, sits at Tier C without causal validation, so that “most cell-resolved” and “closest to application” are not the same property (Table 4).

## 5. Spatial Omics: From Two-Dimensional Sections to Thick Tissues

**Figure 4:**
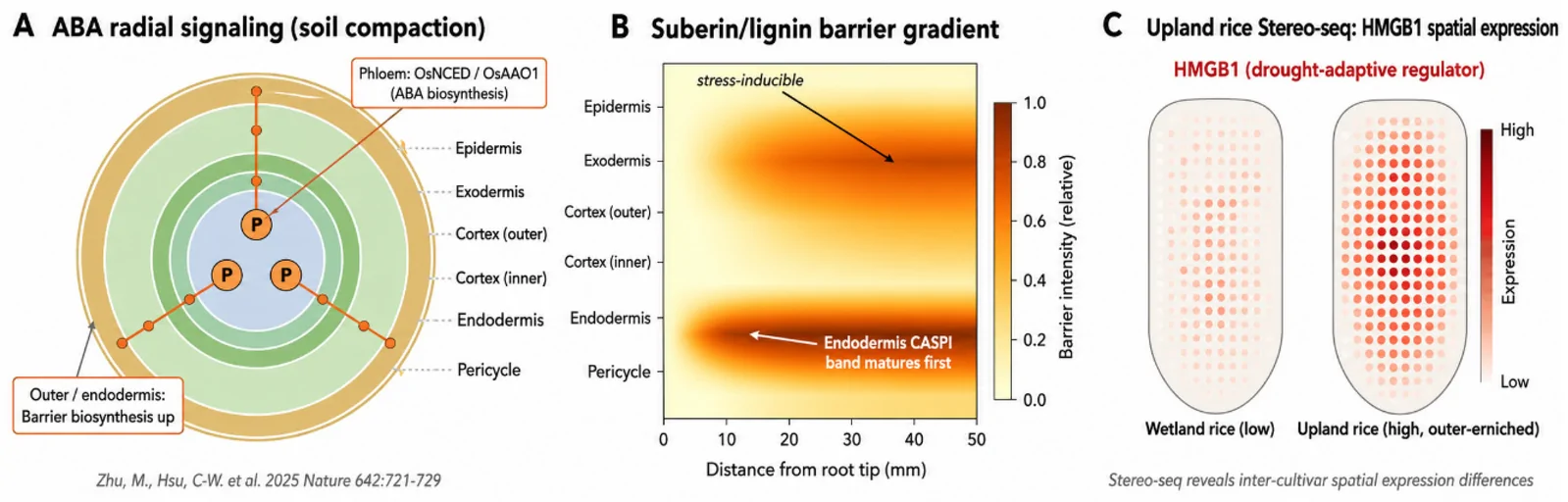
Spatial omics reveals spatial gradients in stress responses. (**A**) Schematic of ABA radial signalling under soil compaction, based on Zhu, Hsu et al. 2025 [28]: in the root cross-section, concentric coloured rings denote successive tissue layers from the stele outward to the epidermis, the orange circles marked “P” indicate phloem sites of ABA biosynthesis (*OsNCED*/*OsAAO1*), and the small orange dots along the radial lines indicate the outward direction of signalling toward the exodermis and endodermis, where barrier-biosynthesis genes are induced. The pattern is consistent with, but does not by itself prove, radial ABA movement (Section 4.2). (**B**) Schematic integrating published histological and spatial results on the suberin/lignin barrier gradient along the root axis [67,68,88]; colour intensity represents relative barrier intensity (see colour bar), with the endodermal Casparian band maturing earliest and exodermal suberin reinforced under stress further from the tip. This panel is an integrative schematic, not a single primary spatial dataset. (**C**) Upland-rice Stereo-seq showing *HMGB1* enrichment in outer-cell layers relative to lowland rice [34]; each dot is a spatial spot, and dot colour and intensity encode *HMGB1* expression level (from pale, low, to dark red, high; see legend). Panels A and C summarize primary spatial studies (Section 5.1 and Section 5.3); panel B is an integrative schematic.

Dissociation-based methods discard position; spatial omics recovers it, and for rice the recovered information determines whether an intervention should target the epidermis, endodermis, vasculature, or a defined axial zone. This section separates results demonstrated directly in rice from applications that are plausible extensions of demonstrated methods, and adds a treatment of the three-dimensional approaches needed for thick plant organs. Figure 4 presents representative rice results.

### 5.1. Radial Signalling and Barrier Gradients: Direct Rice Evidence

The soil-compaction study of Zhu, Hsu et al. is the most complete rice abiotic stress spatial study [68]. Integrating scRNA-seq of over 47,000 cells with spatial transcriptomics, it localized ABA-biosynthesis genes to phloem-associated vasculature and barrier-biosynthesis genes to exodermis and endodermis, which showed the highest differential expression of any cell type. Barrier deposition also follows a longitudinal gradient, the endodermal Casparian band maturing earliest [65,89] and exodermal suberin reinforced under stress further from the tip [66]. Spatial omics contributes here what histology cannot: genome-scale transcriptomic dynamics captured simultaneously across all layers and axial positions. As Section 4.2 stressed, the appropriate causal language is that the pattern is consistent with radial ABA movement, not that it demonstrates it.

### 5.2. Cell–Cell Communication and Local Microenvironments

Spatial data also identify local functional units. The clearest rice example is biotic: combining leaf snRNA-seq and spatial transcriptomics during blast infection, roughly 75% of single-cell differentially expressed genes were undetectable in bulk data, and cells at infection sites responded ahead of their neighbours [88]. This is the most concrete available argument that whole-tissue averaging discards cell-type-specific signal, and it plausibly extends to spatially uneven abiotic stress—patchy salinity, localized drying, uneven Cd. But it is a methodological analogy, not abiotic stress evidence: the result was obtained for a biotic stress in leaf, and no equivalent abiotic stress measurement exists in rice. The 75% figure should be cited as motivation, not as support for an abiotic claim.

### 5.3. Cross-Cultivar Spatial Comparison

The upland-rice root Stereo-seq study is the first cross-cultivar spatial comparison in rice, finding *HMGB1* enriched in outer cells of upland relative to lowland rice [37]. This design—asking which expression differences distinguish adapted from unadapted germplasm and in which cells—is the one most directly aligned with breeding. Its limit is intrinsic to two-cultivar comparison: it produces candidates, not causal effects, because the thousands of background differences between two genotypes cannot be separated. Advancing *HMGB1* from a spatial difference to a breeding candidate would require near-isogenic lines, multiple tolerant and sensitive backgrounds, replicated field phenotyping, and functional validation.

### 5.4. Applications Not Yet Demonstrated in Rice

Four uses follow from demonstrated methods but have not been performed in rice, and are stated as testable propositions: pairing Stereo-seq with elemental imaging to map where along the radial path Na^+^ or Cd is retained relative to transporter expression [83,85]; resolving perivascular loading and unloading; profiling the guard-cell/subsidiary-cell microenvironment under heat and water deficit; and localizing the anther tapetal response under high temperature, presently the largest gap between an important trait and available cell-resolved evidence.

### 5.5. Computational Integration

Spatial data require deconvolution [50,51], reference mapping [52], and, increasingly, graph-based methods for spatial-domain identification and neighbourhood analysis [90,91]. Given the scarcity of rice spatial data, cross-species and cross-cultivar integration is common and demands attention to batch effects; deconvolution accuracy depends on the single-cell reference, so Visium-based cell-type claims inherit the recovery biases of Section 2.

### 5.6. Toward Three-Dimensional Spatial Omics in Thick Plant Tissues

Nearly all plant spatial transcriptomics is two-dimensional, yet the organs of greatest interest for stress biology—mature roots, vascular bundles, nodes, anthers—are volumetric structures whose radial and longitudinal organization a single section captures only partially. Three routes toward volumetric analysis exist, at very different maturity levels, and the distinctions matter for what can honestly be claimed.

The first is serial-section reconstruction: acquiring adjacent 2D spatial sections and computationally registering them into a volume. This is the most accessible route and has been demonstrated in animal tissue, where methods now assemble registered 3D blocks from serial sections [92]. Its costs are practical—section deformation and registration error accumulate through the stack—and no rice application has been published, so for rice this is a proposal, not a capability.

The second is tissue clearing combined with in situ imaging, in which a whole organ is rendered optically transparent and target transcripts or proteins are imaged through depth. Clearing chemistry is established in plants, with ClearSee and its derivatives permitting deep fluorescence imaging of intact roots and reproductive organs [41]. This route suits validation of a small number of candidates in three dimensions rather than genome-scale discovery, and its throughput is correspondingly limited. Expansion microscopy, which physically enlarges a specimen to gain effective resolution [93], has now been demonstrated in intact plant root tissue [94], establishing that the approach works despite the cell wall—a genuine positive existence result, though again at the scale of targeted imaging rather than whole-transcriptome profiling.

The third—true volumetric spatial transcriptomics of the kind STARmap achieves in animal tissue [40]—has not been demonstrated in any plant. Cell walls impede probe penetration through depth, autofluorescence compounds with tissue thickness, and no plant volumetric transcriptomic dataset exists. A plant-focused analysis concluded that three-dimensional spatial molecular analysis in plants is presently achieved only by stacking two-dimensional sections and remains an integration aspiration rather than a realized method [95]. For rice specifically, the honest statement is that thick-tissue spatial omics is a direction with clear anatomical motivation—mature roots, nodes, and anthers would all benefit—but with cell wall, autofluorescence, and probe-penetration bottlenecks unresolved, and with no volumetric rice dataset yet in existence.

## 6. Multi-Omics Integration Beyond Transcriptomics

The preceding sections concern RNA and, through multiome, chromatin. A review titled for “omics” must address whether transcriptional observations correspond to protein abundance, post-translational modification, or metabolite accumulation, and how transcriptome, chromatin, methylome, proteome, metabolome, and genetic variation jointly support causal inference. The organizing question of this section is therefore not “what other omics exist” but “which layers can corroborate or contradict a single-cell transcriptomic finding in rice, and at what maturity.” For each layer we state plainly how far plant capability actually extends.

### 6.1. Transcriptome–Chromatin Integration

The most mature integration is transcriptome with chromatin accessibility, now co-assayed per nucleus by multiome [23] and separately by scATAC-seq [9,10,56]. The value is that an expression change can be linked to an accessible regulatory region and thence to a candidate promoter or cis-element. The essential caution, repeated because it is routinely elided, is that open chromatin indicates regulatory potential only: accessibility does not entail transcription, and neither entails a causal effect on phenotype. Multiome strengthens candidate nomination but does not, by itself, close the inferential gap from correlation to cause.

### 6.2. Single-Cell and Single-Nucleus DNA Methylome

Abiotic stress tolerance in plants is linked to epigenetic regulation, including DNA methylation of transposable elements and stress-responsive loci, and methylation is a candidate carrier of stress memory. In rice, multigenerational drought imposition produced heritable methylation changes, a substantial fraction transmitted across generations [96,97]. This evidence is, however, both bulk-level and contested: in *Arabidopsis*, the methylome was reported largely stable under transgenerational drought [98], so the field genuinely disagrees about how much stress memory methylation carries, and a review should represent that disagreement rather than assert the memory model.

The methodological statement that matters most here is an existence claim: single-cell or single-nucleus DNA methylome profiling has not been demonstrated in any plant species. The methods that would be required—single-cell bisulfite sequencing [99], single-nucleus methylcytosine sequencing [100,101], combinatorial-indexing methylation [102], and joint methylome-plus-transcriptome assays [103]—were all developed and applied in mammalian systems. The animal maturity cannot be transferred to rice by assertion; the cell-wall dissociation problem, low per-cell DNA input, and absence of plant-optimized protocols all remain. This defines a concrete opportunity rather than an available tool: whether, in tolerant-versus-sensitive rice, methylation differences in exodermis, endodermis, and vascular tissue correspond to differences in ion transport or barrier formation is a well-posed and currently unanswerable question, because the single-cell methylome measurement does not yet exist for plants.

### 6.3. Complementary Proteomics and Metabolomics

The function of these layers in this review is corroboration, not survey: they test whether transcriptional changes propagate to protein and metabolite. A transcript change need not produce a protein change, so single-cell or spatial proteomics can confirm or refute that a cell-type transcriptional signature reaches the protein level; phosphoproteomics can add the kinase-signalling dimension that transcriptomics misses; and metabolomics, ionomics, and mass spectrometry imaging can localize the actual effectors—ABA, osmolytes, Na^+^, Cd, As—within tissue.

Maturity must again be stated honestly. Mass-spectrometry single-cell proteomics methods [104,105,106] are mammalian; in plants, single-cell proteomics is at proof-of-concept, with the rigid cell wall and picogram-scale per-cell protein input as primary bottlenecks, and no routine plant workflow exists [107]. Plant single-cell metabolomics has genuine but low-throughput demonstrations—live single-plant-cell mass spectrometry [108]—and spatial metabolomics by mass spectrometry imaging has been applied to a cereal under abiotic stress, mapping metabolites and lipids in barley roots under salinity [109], which is the closest existing analogue to what rice work would require. The accurate summary is that in rice these modalities remain largely tissue- or region-scale and are not yet mature single-cell or spatial technologies; they can corroborate transcriptomic findings today only at coarse resolution, and their single-cell counterparts are years behind transcriptomics.

### 6.4. Genetic and Phenotypic Integration

**Figure 5:**
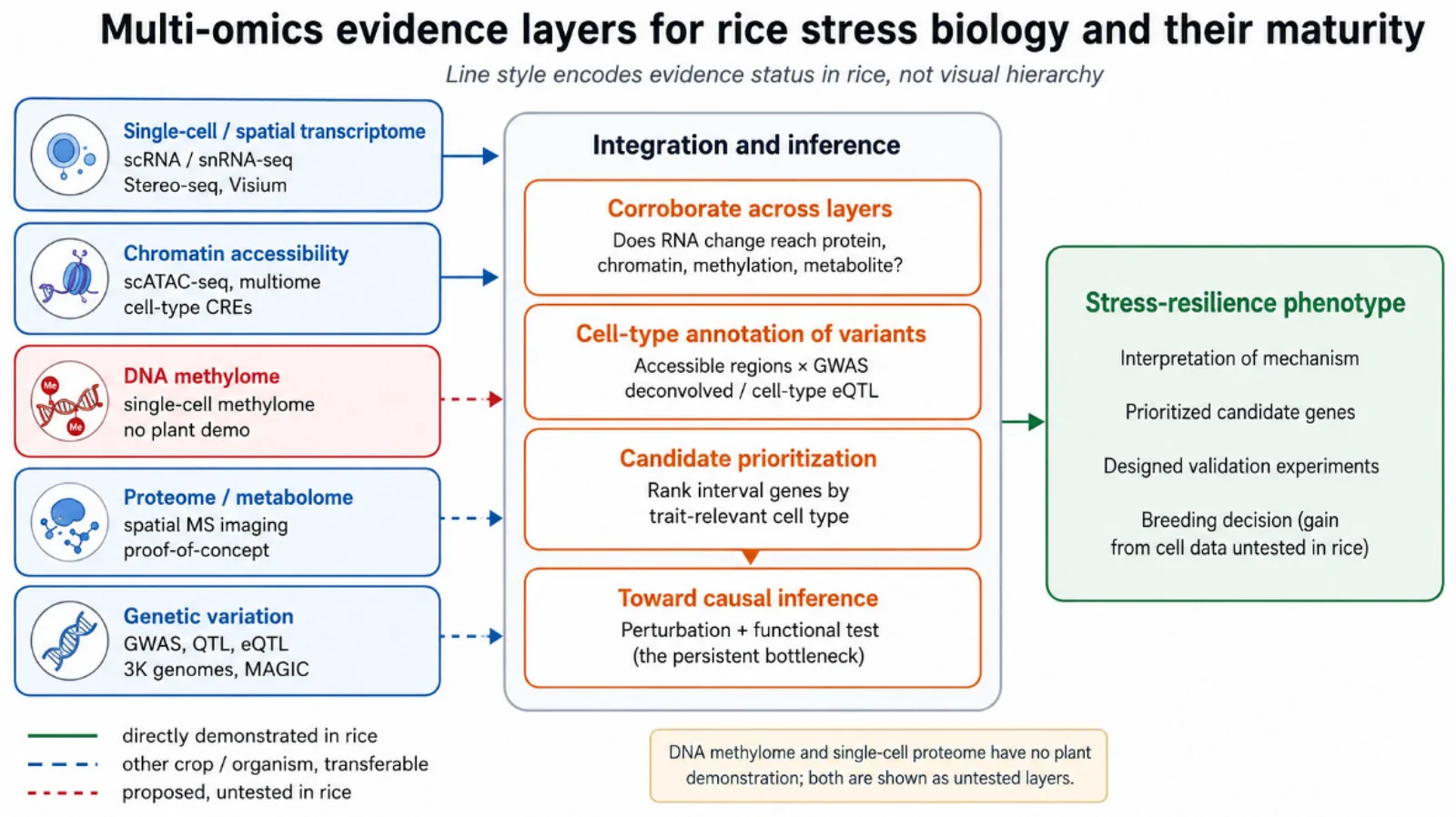
Multi-omics evidence layers for rice stress biology and their maturity in rice. Input layers — single-cell/spatial transcriptome, chromatin accessibility, DNA methylome, proteome/metabolome, and genetic variation — feed inference steps that connect molecular observation to phenotype. Arrows indicate the direction of information flow, from the input molecular layers on the left, through the integration and inference steps, to the stress-resilience phenotype on the right. The line style of the arrows linking each input layer to the inference steps encodes the maturity of that evidence in rice: solid, directly demonstrated in rice; dashed, demonstrated in other crops or organisms and transferable in principle; dotted, proposed but untested in rice (including single-cell methylome and single-cell proteome, which have no plant demonstration). The figure summarizes published evidence and does not propose a new framework.

The final layer connects molecular observation to heritable variation and phenotype. Cell-type-specific eQTL, established in human blood [110], is early in crops; the practical rice route is bulk-population eQTL on resources such as the 3000 Rice Genomes [111] and MAGIC populations [112], with single-cell atlases used to attribute bulk signals to cell types [113,114]—cell-type attribution of a bulk signal, not direct cell-type-specific measurement. Intersecting cell-type accessibility with GWAS intervals [23,115,116] converts a mapped interval into a hypothesis about where a variant acts. This layer should be understood as a methodological chain from multi-omic association toward functional validation, not as an established breeding pathway: each step—accessible, therefore differentially expressed, therefore causal—is an inference requiring its own test. Figure 5 summarizes the layers and, by line style, the evidence status of each linkage.

## 7. Translational Implications

Having treated the methods and the evidence they generate, this section asks what that evidence currently supports downstream, distinguishing throughout what has been validated in rice from what remains methodological prospect. It is deliberately shorter than the methodological core, because the central message is a boundary rather than a roadmap.

### 7.1. Candidate Prioritization

The most concrete current contribution of single-cell atlases is prioritization. A GWAS or QTL interval holds tens to hundreds of linked genes, and with 40–50% of rice genes lacking informative functional annotation, annotation-driven ranking is limited. Cell-type specificity adds an orthogonal filter: within an interval, a candidate expressed in a mechanistically plausible cell type—stelar parenchyma for xylem Na^+^ retrieval, exodermis for barrier traits, tapetum for pollen viability—ranks above one expressed only in unrelated tissue, and this filter is strongest exactly where annotation fails, because specificity is measured directly. The demonstrated rice case is *OsLTPL120*, nominated from root-epidermis-specific expression and confirmed by a mutant phenotype [23]. That case shows the logic works; it does not show a gain in hit rate, because no published comparison exists between cell-type-informed and annotation-driven prioritization on matched intervals, and *OsLTPL120* was validated for a developmental, not a resilience, phenotype. A fair future test would report, on a common set of intervals, the precision-at-k, validation success rate, per-positive validation cost, and field effect size of cell-type prioritization against annotation-based ranking.

### 7.2. Interpreting Mapped Loci and Designing Experiments

Cell-type information clarifies why established loci work—*OsHKT1;5* is comprehensible only because its transporter sits in xylem-adjacent stelar parenchyma [5], *OsHMA3* because it acts at the root vacuolar membrane [81,82]—and the accessibility-by-GWAS intersection [23] scales this interpretation across many intervals. It can also guide phenotyping: a cell-layer-proximal phenotype such as exodermal suberin deposition or tapetal degeneration timing may, in principle, have simpler genetic architecture than whole-plant yield under stress, though this expectation is itself a hypothesis requiring direct heritability comparison rather than an established result. The persistent limit is that chromatin accessibility in a cell type is several inferential steps from causality, and that *indica*–*japonica* divergence [111,116] means annotation from a *japonica* reference may not transfer to *indica* material.

### 7.3. Functional Validation and Engineering: An Evidence Matrix

The clearest way to represent the translational state is as a matrix of readiness. Deployed, from classical genetics and field validation: *SUB1A* [6], *OsHKT1;5* [5], *OsHMA3* [81], *OsNRAMP5* [87], *DRO1* [67]. Cell-localized but awaiting functional validation: *HMGB1* [37], the exodermal MYB/NAC suberin module [22]. Methodological proposal only*:* cell-type-specific promoters, cis-regulatory editing, and GRN-guided gene stacking. The cell-type-specific promoter strategy has proof-of-concept in *Arabidopsis*—cortex-specific *AtHKT1;1* [63]—and the multi-organ and *Poaceae* atlases supply candidate cell-type-specific accessible regions as raw material [23,31], but very few rice cell-type-specific promoters are validated across backgrounds, and accessibility in an atlas does not guarantee faithful expression from an isolated fragment. Cis-regulatory editing is feasible in rice [117] and has quantitative-trait proof-of-concept in tomato [118], with the *Poaceae* atlas identifying silencer-like elements whose deletion upregulated targets [31]; its limit is predictability across backgrounds. GRN-guided stacking remains an argument, not a rice demonstration. The matrix makes the review’s central asymmetry explicit: everything in the “deployed” row reached the field without single-cell data.

### 7.4. Genomic Prediction: A Testable Statistical Hypothesis

Prediction under stress is poor because genotype-by-environment interaction is large and causal variants numerous [119]. Weighting the genome by cell-type annotations of stress-relevant regulatory variation might improve stress-trait prediction, and multi-kernel or annotation-weighted GBLUP and deep-learning models [120,121,122] provide frameworks. But no published test compares cell-type-weighted prediction against GBLUP or BayesB baselines on rice stress-trial data, and functional-annotation weighting in plants has given small, inconsistent gains [123]. The honest status is a well-posed, untested hypothesis: there is at present no evidence that cell-type-weighted models outperform standard baselines in rice, so this should be presented as a statistical hypothesis to be tested—with independent atlas-derived annotations, realistic prediction into untested lines and environments, and variance reported across replicates [111,112]—not as a realized advantage. Cell-type annotation’s one practical attraction is that, unlike per-individual transcriptomes, it is generated once and reused at no per-individual cost.

## 8. Challenges and Future Directions

**Figure 6:**
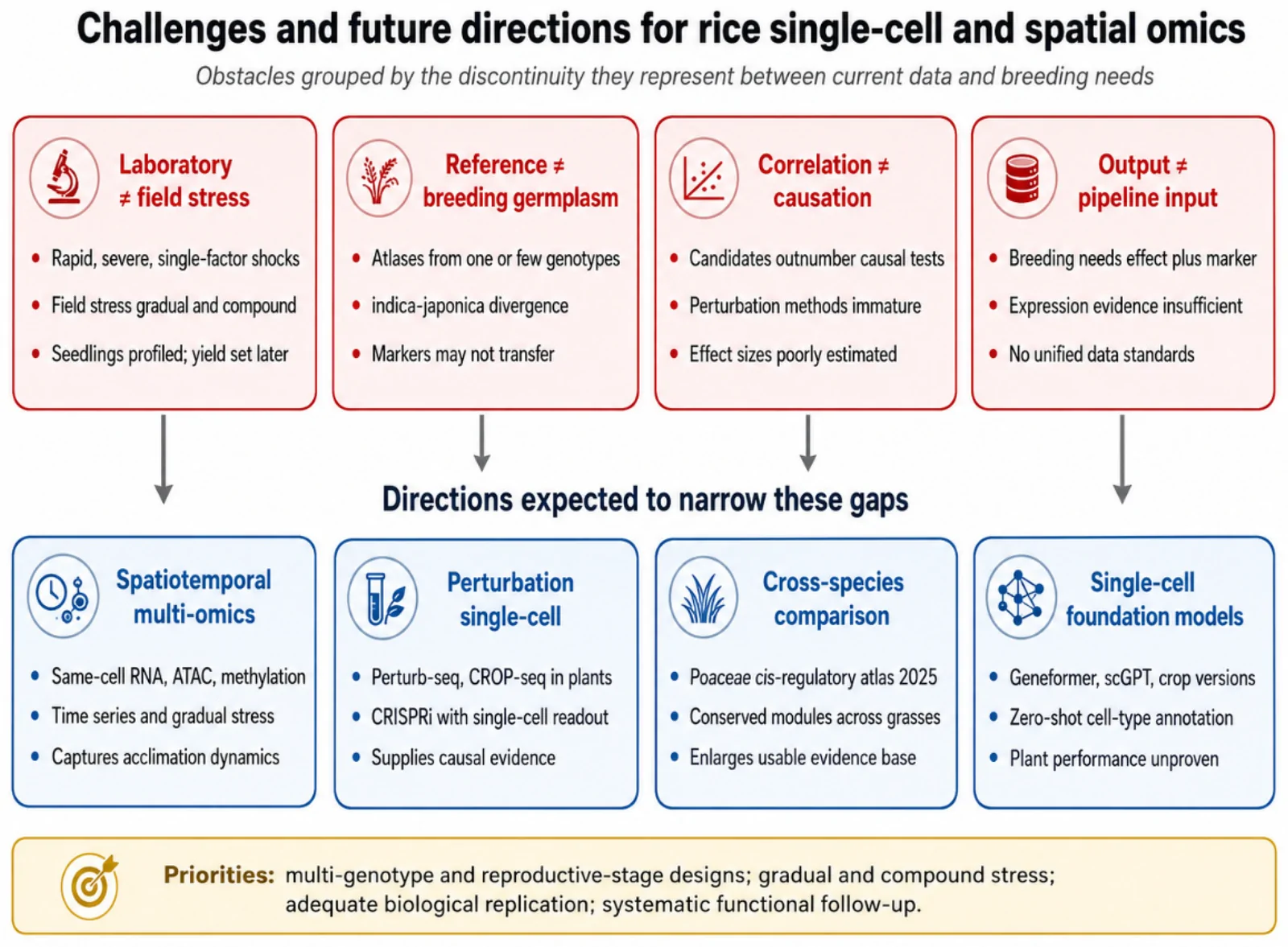
Challenges and future directions for rice single-cell and spatial omics, grouped by the discontinuity each represents—laboratory versus field stress, reference versus breeding germplasm, correlation versus causation, and research output versus breeding pipeline—with the technical and analytical directions expected to narrow them, including the artificial-intelligence tasks detailed in Section 8.8.

Rice single-cell and spatial studies now provide organ atlases, stress-responsive cell profiles, and regulatory hypotheses. Three limitations recur across the literature: technical bias introduced during tissue preparation, incomplete biological validation of inferred cell states and networks, and limited evidence connecting cell-level measurements to field traits. Figure 6 summarizes these constraints and the experiments needed to address them.

### 8.1. Laboratory Versus Field Stress

Nearly all rice single-cell stress data come from rapid, severe treatments on seedlings in controlled culture. Field stress develops gradually, permitting acclimation and shock bypass; is compound; is spatially and temporally heterogeneous; and acts at yield-determining reproductive stages [3,29] rather than the seedling stage almost always profiled. A cell type maximally responsive to a laboratory shock need not be the one limiting field performance. Bridging requires field or field-simulating experiments with snRNA-seq on flash-frozen material—feasible now [8] but rarely done.

### 8.2. Reference Versus Breeding Germplasm

Atlases derive from one or a few *japonica* references, while breeding germplasm is diverse; *indica*–*japonica* divergence is large enough that regulatory-element presence, expression pattern, and marker reliability can differ [111,116], so a reference-genotype annotation cannot be assumed to hold in elite *indica* or landrace donors without checking. Developmental coverage is equally narrow—seedling root tips dominate—leaving the reproductive, yield-critical windows least profiled.

### 8.3. Correlation Versus Causation

Single-cell studies produce associations; breeding needs causal claims of defined magnitude in defined backgrounds. Most current candidates—*OsLTPL120*, *RSR1*, *HMGB1*, *OsF3H*, exodermal MYB/NAC members—have not crossed that distance. Closing it requires perturbation: adapting Perturb-seq [124] and CROP-seq [125] to plants, coupling CRISPR-interference libraries to single-cell readout. Plant applications remain limited by transformation throughput, but progress here would convert candidate lists into breeding material faster than any further enlargement of atlases.

### 8.4. Technical and Cost Constraints on Design

Protoplasting bias is best mitigated by snRNA-seq [8]; where unavoidable, transcription inhibition and fixation reduce artefacts [16]. Rice-specific markers remain sparse for the multilayered cortex, aerenchyma, and transitional states [47]. Cost most directly limits design: high per-experiment cost forces few genotypes, conditions, timepoints, and replicates, so effect sizes are poorly estimated, and cross-study comparison is confounded [53]. Combinatorial indexing (Section 2.3) is the most direct structural response, trading per-cell depth for the multiplexing that adequate replication and multi-genotype contrasts require.

### 8.5. Standards and the Pipeline-Entry Threshold

The field lacks unified formats, cell-type nomenclature, and analytical standards, limiting meta-analysis. A less-discussed barrier is the threshold for entering a breeding pipeline: with multi-year cycles and fixed resources, a new target displaces an existing one, and cell-type-specific expression alone does not justify displacement. The practical requirements—a validated effect in a relevant background, a field-detectable effect size, an acceptable trade-off profile, and a marker or editing route—are met by almost no current cell-resolved candidate. Recognizing this threshold argues for designing studies with tolerant and susceptible contrasts from the outset.

### 8.6. Toward a Unified Rice Single-Cell and Multi-Omics Stress Atlas

A recurring practical question is whether the scattered data surveyed here could be consolidated into a single rice atlas with integrated visualization, in the manner that the Klepikova developmental transcriptome [126] is rendered for *Arabidopsis* and that “electronic fluorescent pictograph” browsers provide for painting expression onto tissue schematics [127]. The building blocks exist but are siloed by modality. Rice bulk and stress transcriptomes are well served by dedicated resources—RiceXPro for spatiotemporal field expression [128], TENOR for mRNA-seq across ten abiotic stress and two hormone conditions [129], and RED for integrated RNA-seq [130]. Single-cell and chromatin data are curated by multi-species platforms—scPlantDB [47], PsctH [48], PlantscRNAdb [49]—in which rice is one species among many, and by the dedicated portals of the two 2025 rice single-cell atlases [23,31]. Genomic, proteomic, and metabolomic layers reside in still further separate resources. Critically, no current resource unifies single-cell, spatial, transcriptome, translatome, proteome, and metabolome data for rice under stress in one atlas with common visualization, and the single-cell products that do exist are largely non-stress and RNA- or chromatin-only. Building such a resource is therefore an unmet need rather than a solved problem. It would require a warehousing-and-linking architecture of the kind articulated for the Plant Cell Atlas [131], a standardized cell-type ontology and harmonized stress-condition and multi-omics metadata [132], and dual visualization—pictograph-style rendering for tissue-level layers and UMAP-style embedding for single-cell layers. Developing an integrated, stress-aware rice atlas and analysis platform along these lines is a concrete and worthwhile direction for the field.

### 8.7. Extending Beyond Transcriptomics

The modality gaps of Section 6 are themselves future directions: no plant single-cell methylome, no mature plant single-cell proteomics, no volumetric plant spatial transcriptomics. Progress on any would enlarge the evidence base, but each faces the shared plant obstacles of cell wall, autofluorescence, and low per-cell input, and none should be presented as imminent.

### 8.8. Artificial-Intelligence-Enabled Analysis of Large, Multimodal Plant Datasets

Artificial intelligence enters this field not as a single tool but as a set of task-specific methods at differing maturity, and Figure 6’s AI element stands for the following concrete tasks, each with its own inputs, outputs, validation requirements, and failure modes.

Cell-type annotation and cross-species mapping: Supervised and reference-based models transfer labels from annotated atlases to new datasets; foundation models such as scGPT [133], Geneformer [134], Universal Cell Embeddings [135], and scFoundation [136] propose zero-shot annotation and cross-dataset integration from large pretraining. For plants, the relevant and genuinely positive development is scPlantLLM, a plant-specific single-cell foundation model reporting high zero-shot annotation accuracy on *Arabidopsis* [137]—distinct from plant DNA language models such as AgroNT [138] and PlantCaduceus [139], which model genomic sequence rather than single-cell state. The input is an expression matrix; the output is per-cell labels or embeddings; the validation requirement is agreement with independently established markers, especially for rice cell types absent from mammalian-dominated pretraining.

Image segmentation and three-dimensional reconstruction: Deep-learning segmentation—Cellpose [140], StarDist [141]—and the plant-adapted PlantSeg [142] delineate cells and nuclei in tissue images, a prerequisite for imaging-based spatial methods and for the serial-section and cleared-tissue 3D routes of Section 5.6. In rice, irregular, tightly packed cells and cell-wall autofluorescence make plant-tuned models necessary; the validation requirement is manual ground truth on the specific tissue, since models trained on animal images transfer imperfectly.

Batch correction, spatial-domain identification, and neighbourhood analysis: Integration methods [42,43] remove technical batch structure, and graph neural networks [90,91] identify spatial domains and cell neighbourhoods from spatial data. The failure mode is specific and consequential: aggressive batch correction can remove genuine biological differences between genotypes or treatments along with technical artefacts, so in a tolerant-versus-sensitive comparison the correction step can erase the signal of interest, and its strength must be validated against known controls.

Multi-omics integration and GRN inference: Models that align transcriptome, chromatin, and, in principle, methylome or protein layers, and that infer gene regulatory networks from these, are how Section 6 layers would be computationally joined. Inferred networks are hypotheses: edge predictions require perturbation validation, and networks inferred from one genotype under control conditions may not hold under stress or across backgrounds.

Perturbation-effect prediction: Foundation models are increasingly proposed to predict the effects of genetic perturbations in silico, which would prioritize edits before experiment. This is the least mature application and the most consequential to assess honestly.

Risks, stated as they are documented: Four risks are not speculative but demonstrated. First, mammalian training bias: the major foundation models are pretrained overwhelmingly on animal data, and plant cell-type diversity, polyploidy, and regulatory architecture differ, so transfer is not guaranteed, and scPlantLLM’s recency and singularity underline how thin plant-specific pretraining still is. Second, and most important, rigorous benchmarks show that single-cell foundation models frequently fail to beat simple baselines: zero-shot scGPT and Geneformer performed inconsistently against embeddings from highly variable genes, scVI, or Harmony [143], and a logistic-regression baseline matched or outperformed scBERT even in few-shot settings [144]. Third, black-box inference: high-dimensional models yield predictions whose basis is opaque, which is hazardous when the output is a candidate gene or edit rather than a classification. Fourth, reference–atlas bias: models inherit the coverage and annotation biases of their training atlases, so a cell type or state under-represented in rice references will be under-served regardless of model capacity. The through-line is that AI accelerates analysis but does not supply biological validation; every AI output relevant to a breeding decision—an annotation, a network edge, a predicted perturbation effect—requires independent experimental confirmation, and the benchmarking literature counsels comparing any foundation-model result against a simple baseline before accepting it.

## 9. Conclusions and Perspectives

Single-cell and spatial omics have given rice a usable cell-type reference for the major organs and have resolved several stress responses to specific cell populations with direct rice evidence: ABA-associated radial signalling to exodermis and endodermis under soil compaction [68], cell-type-specific chromatin remodelling under heat [56], and suberin-barrier regulation under water deficit [22]. Cross-cultivar spatial comparison has been demonstrated [37], and cell-type accessibility has been intersected with GWAS signals to yield cell-type-by-trait associations with at least one candidate followed to a validated mutant phenotype [23].

Read through the evidence-tier scheme, the field’s central asymmetry is clear. The genes with genuine breeding traction—*SUB1A* [6], *OsHKT1;5* [5], *OsHMA3* [81], *OsNRAMP5* [80,87], *DRO1* [67]—rest on Tier B classical genetics and field validation, and every one reached the field without single-cell data. The richest cell-resolved rice evidence, in root outer-layer barrier programmes, sits at Tier C without causal validation. Heat and cold stress, which dominate yield loss, are almost devoid of rice cell-resolved data, and the anther heat-stress single-cell study that reproductive thermotolerance most needs does not exist. Extending the field’s own title to “omics,” the methylome, proteome, and metabolome layers are at plant maturities well below transcriptomics: no plant single-cell methylome, proof-of-concept-only plant single-cell proteomics, and region-scale rather than single-cell plant spatial metabolomics.

Progress therefore depends less on larger atlases than on better-designed experiments: multiple genotypes with tolerant and susceptible contrasts, gradual and compound stress, reproductive-stage tissues, adequate biological replication—for which combinatorial indexing is now the enabling method—and systematic functional follow-up through plant-adapted perturbation approaches. Complementary layers should be added where they can corroborate transcriptomic findings, and artificial-intelligence methods should be used for what they demonstrably do—annotation, segmentation, integration—while every downstream biological claim is validated independently and benchmarked against simple baselines.

To date, cell-resolved omics has primarily improved biological interpretation and candidate prioritization, whereas the strongest breeding successes in rice still rest on classical genetic validation and field testing. Demonstrating an incremental breeding advantage from cell-resolved information—a candidate reaching the field, or a prediction model outperforming standard baselines, because of it—remains the central unmet requirement, and meeting it is the measure by which the coming decade of this technology should be judged.

## Figures and Tables

**Table 1:** Published rice single-cell and spatial omics studies (through early 2026).

Study (Year)	Organ	Platform	Cells	Cell Types	Stress	Key Findings
Zhang T-Q et al. 2021 [20]	Root tip	scRNA + scATAC	~27k	9	None	Rice root atlas with chromatin accessibility
Liu Q et al. 2021 [24]	Root	scRNA-seq (10×)	~21k	10	None	Root developmental trajectory and cell-type markers
Wang Y, Qian W 2021 [21]	Seedling (leaf + root)	scRNA-seq	237k	24	Salt/low N	Large-scale rice seedling stress atlas
Zong J et al. 2022 [25]	Young panicle	scRNA-seq	~25k	8+	None	Panicle development and spikelet differentiation
Feng D et al. 2022 [26]	Root tip	scATAC-seq	46.8k	-	Heat	Cell-type-specific ACRs and heat response
Reynoso et al. 2022 [22]	Root	INTACT + TRAP + ATAC	-	-	Water extremes	Cell-type GRNs and suberin-related regulation
Li C et al. 2023 [27]	Pistil	snRNA-seq	~15k	-	None	Pistil developmental trajectories
Yao J et al. 2024 [28]	Embryo	Stereo-seq + scRNA	-	-	None	Embryo germination atlas and scutellum cell states
Zhu M, Hsu C-W et al. 2025 [29]	Root	scRNA + Molecular Cartography	>47k	-	Soil compaction	ABA-associated vascular-to-outer-tissue signalling
Wang X et al. 2025 [23]	8 organs	10 x Multiome	116k	multiple	None	Rice multi-organ single-cell multi-omics atlas
Yan H et al. 2025 [30]	Rice + Poaceae	snRNA + scATAC	104k	126 states	None	Cross-species cis-regulatory evolution
Wang W et al. 2025 [31]	Leaf	snRNA + spatial	236k+	-	Blast infection	Stereoscopic response pattern (biotic-stress analogy)

Note: Cell-type numbers are approximate where the original paper did not report exact counts; “-” indicates not reported.

**Table 2:** Comparison of single-cell and spatial omics platforms.

Platform	Resolution	Throughput	Spatial	Plant Compatibility	Cost	Representative Rice Use
scRNA-seq (10×)	Single cell	High (10^4^–10^5^)	No	Needs protoplasts; stress artefacts possible	Medium	Zhang 2021 [20], Wang/Qian 2021 [21]
snRNA-seq	Single nucleus	High (10^4^–10^5^)	No	Low stress; hard-to-dissociate cells	Medium	Multi-organ atlas and pistil atlas
scATAC-seq	Single nucleus, chromatin	Medium (10^4^)	No	Suitable; complements scRNA	Medium-high	Root heat-stress chromatin atlas
10× Multiome	Single nucleus RNA + ATAC	Medium (10^4^)	No	Suitable	High	Wang X 2025 Nature [23]
Stereo-seq	Subcellular-scale	High, large FOV	Yes	Suitable with tissue optimization	High	Rice embryo germination
Molecular Cartography	Targeted, near-cellular	Probe-panel dependent	Yes	Requires tissue-specific probes and imaging	High	Rice root soil compaction
MERFISH/seqFISH	Subcellular	Targeted gene panels	Yes	Plant use remains technically demanding	High	Nascent for crop applications

**Table 3:** Single-cell and spatial omics across rice, maize, and wheat.

Dimension	Rice	Maize	Wheat
Technology maturity	Root, seedling, and multi-organ atlases relatively rich	Root, leaf, and developmental studies active	Single-nucleus strategies predominate
Principal difficulty	Exodermis, mature roots, field material	Tissue complexity, genotype diversity	Allohexaploidy, ~17 Gb genome, homoeolog and annotation difficulty
Abiotic stress gap	Salt-stress root, anther heat stress	Cross-tissue and field-stress validation	High-quality cell annotation under stress
Lessons for rice	Needs more spatial and multi-genotype studies	Cross-species regulatory atlases	Single-nucleus and homoeolog-aware analysis for cross-subspecies and polyploid donors

**Table 4:** Rice resilience genes and candidates by cell type, evidence tier, and breeding status.

Gene/Module	Stress	Key Cell Type	Evidence Tier	sc/Spatial Evidence	Breeding Status
*SUB1A*	Flooding	Node, internode	B (+field scale)	Only recently addressed	Deployed in mega-varieties
*OsHKT1;5*/*SKC1*	Salt	Stelar parenchyma	B	Promoter-reporter; no sc study	Deployed in MAS
*OsHMA3*	Cd	Root vacuolar membrane	B	Inferred	Allele selection, low-Cd rice
*OsNRAMP5*	Cd/Mn	Epidermis, exodermis	B	Immunolocalization	Editing, near-release lines
*DRO1*	Drought	Root tip	B (+field yield)	Not resolved	Field-verified benefit
*OsLsi1*/*OsLsi2*	As/Si	Exodermis, endodermis	B	Polar localization shown	As mitigation
*COLD1*, *HAN1*, *bZIP73*, *CTB4a*	Cold	Not resolved	B	None	Usable in MAS
*TT1*/*TT2*/*TT3*	Heat	Not resolved	B	None	Donor alleles available
*qHTSF4.1*	Heat (anthesis)	Anther tapetum (presumed)	B	None	Donor N22 in breeding
MYB/NAC suberin module	Drought, salt	Exodermis, endodermis	C	sc + spatial	Promoter-restricted-expression target
*OsNCED*/*OsAAO1*	Compaction/drought	Phloem vasculature	C (spatial)	Direct spatial evidence	Signalling source
*HMGB1*	Drought (upland)	Root outer layers	C (spatial)	Cross-cultivar spatial	Candidate; causality untested
*OsLTPL120*, *RSR1*, *OsF3H*	Not defined	Epidermis; meristem; multiple	C	sc	Candidates only
Cortical heat response	Heat	Root cortex	C (rice)/D (conservation)	scATAC (rice); RNA (maize)	Not yet a target
Cortex-specific *AtHKT1;1*	Salt	Root cortex	D (*Arabidopsis*)	n/a	Proof-of-concept; untested in rice

## Data Availability

No new data were created or analyzed in this study. All data and findings discussed in this review are derived from previously published studies cited in the References.
